# Gene profiles and mutations in the development of cataracts in the ICR rat model of hereditary cataracts

**DOI:** 10.1038/s41598-023-45088-1

**Published:** 2023-10-24

**Authors:** Masaru Takashima, Kei Taniguchi, Masaya Nagaya, Shunki Yamamura, Yoshihiro Takamura, Masaru Inatani, Masaya Oki

**Affiliations:** 1 Department of Industrial Creation Engineering, Graduate School of Engineering, University of Fukui, Fukui, Japan; 2 Department of Ophthalmology, Faculty of Medical Sciences, University of Fukui, Fukui, Japan; 3 Life Science Innovation Center, University of Fukui, Fukui, Japan

**Keywords:** Molecular biology, Medical research

## Abstract

Cataracts are opacifications of the lens that cause loss of visual acuity and ultimately of eyesight. Age-related cataract develops in most elderly people, but the mechanisms of cataract onset are incompletely understood. The Ihara Cataract Rat (ICR) is an animal model of hereditary cataracts showing cortical opacity that commonly develops prematurely. We identified putative mechanisms of cataract onset in the ICR rat model by measuring gene expression changes before and after cortical cataract development and conducting point mutation analysis. Genes differentially expressed between 4-week-old animals without cortical cataracts and 8–10-week-old animals with cortical cataracts were selected from microarray analysis. Three connections were identified by STRING analysis: (i) Epithelial-Mesenchymal Transition (EMT), including *Col1a2,* and *Pik3r1.* (ii) Lens homeostasis, including *Aqp5,* and *Cpm*. (iii) Lipid metabolism, including *Scd1, Srebf1,* and *Pnpla3*. Subsequently, mutation points were selected by comparing ICR rats with 12 different rats that do not develop cataracts. The apolipoprotein *Apoc3* was mutated in ICR rats. Analyses of gene expression changes and point and mutations suggested that abnormalities in EMT or lipid metabolism could contribute to cataract development in ICR rats.

## Introduction

Cataract is a disease in which progressive opacification of the lens causes loss of visual acuity and ultimately of eyesight^1^. Presently, the only available intervention is surgical replacement with intraocular lens, and the mechanisms of cataract formation has not been elucidated.

Cataracts are classified into three types: nuclear cataracts, posterior subcapsular cataracts, and cortical cataracts^2^. Nuclear cataract develops due to aggregation of the crystallin-constituting lens^3^. Posterior subcapsular cataract is caused by dysregulated migration of lens epithelial cells (LECs)^4^. Cortical cataract opacifications occur due to swelling and collapse of fibers at the cortex^5^.

Induction models initiate cataract formation by drug treatment, while in hereditary cataractous models, cataracts spontaneously develop with aging. The selenite induction cataract model induces development of nuclear cataracts^6^. Diabetic cataract models such as STZ rats^7^ and the related galactose-induced cataract model^8^ develop cortical cataract. Mouse and rat models of hereditary cataract are available. The Emory mouse develops nuclear cataract^9,10^, and the senescence-accelerated mouse (SAM)^11^ develops cortical cataract. The Shumiya Cataract Rat (SCR)^12,13^, Upjohn Pharmaceutical Limited (UPL) rat^14,15^, and Ihara Cataract Rat (ICR)^16^ are commonly used rat models of hereditary cataract. The ICR model is a recessive hereditary cataract rat model that develops cortical cataracts beginning slightly before 7 weeks of age, and nuclear cataracts reach maturity after 12 weeks of age^16,17^. The causative genes for cataract formation in ICR are currently unknown. In the Ihara epileptic rat (IER), which is inbred lines selected from ICR for individuals who develop epilepsy, two loci have been identified as potential inducers of nuclear cataract formation, but the causative genes have not yet been identified^18^.

Induction and hereditary models of cataract have distinct benefits and caveats, and models are selected based on the application. We selected to use a hereditary cataractous model in this study. Unlike induction models, cataracts develop in hereditary models over time similar humans, in which cataract develops with age. This study, we focused on ICR. One cause of cataracts in the ICR model is oxidative stress^19^. Serum lipid peroxide levels dramatically increase in ICR rats at 13 days of age^20^. Laser Raman spectroscopy revealed that in ICR lenses relative to wild-type lenses, the S–S bond increased and the SH group decreased in the lens protein ^21^. Furthermore, saccharification of lens proteins contributes to cataract^22^. In ICR lenses, a specific type of glycosidase activity increases on developing cataracts^23^. Therefore, oxidative stress and lens protein saccharification are likely involved in ICR cataract development. Further, lens Ca^2+^ concentration increases and lens glutathione concentration decreases in lenses with cataract^24,25^. Likewise, in ICR rats, lens Ca^2+^ concentration is increased and glutathione concentration is decreased^26–28^. Moreover, excessive NO advances lipid peroxidation and inhibits Ca^2+^-ATPase in ICR lenses^29^. Increased Ca^2+^ concentration activates the calcium-dependent protease calpain, and consequently α-crystallin, which functions as a chaperone, is degraded^27^. Subsequently, depletion of glutathione, an endogenous antioxidant enzyme, promotes an oxidative environment. The resultant oxidized proteins are more susceptible to calpain proteolytic degradation. The first amino acid residue of Met of α-crystallin is oxidized^30^ and its chaperone activity was decreased by C-terminal truncation of α-crystallin in ICR lenses, which occurs by 12 weeks of age^31^. Further, mass spectrometry imaging analysis revealed that the C-terminal cleaved products of α crystallin accumulated around nuclear cataracts in ICR lenses^32^. Thus, crystallin protein aggregation is caused by decreased chaperone activity due to α-crystallin truncation. Although numerous studies have investigated the role of crystallin in nuclear cataract formation in ICR, the mechanism by which early cortical cataracts develop and concomitant changes in gene expression profiles remains incompletely understood.

Previous IER study, suggests involvement of two distinct genes in cataract formation, in which the role of each gene is time dependent^18^. Linkage analysis revealed that Cataract Ihara 1 (*Cati1*) is the locus on chromosome 8 related to cataract formation, and that Cataract Ihara 2 (*Cati2*) is the locus on chromosome 15 related to the timing of cataract formation. Backcrossing experiment were conducted between IER and Wister-Kyoto (WKY) rat that do not develop cataract and known to abundance of genetic polymorphisms ^33^, defining the alleles originating from the IER as i and the allele originating from the WKY as w. In *Cati1*^*i*^/*Cati1*^*i*^ animals, *Cati2*^*i*^/*Cati2*^*i*^ causes early cataract development, and *Cati2*^*i*^/*Cati2*^*w*^ and *Cati2*^*w*^/*Cati2*^*w*^ cause late cataract development. Nuclear cataracts form in the IER model, and the similarities or differences of this mechanism with that of cortical cataract formation in the ICR model remain unclear.

In this study, we identified a gene group involved in ICR cataract development by analyzing lens gene expression changes using microarray analysis of time series sampling according to cataract progression times. Further, we investigated potential cataract-causing genes in the ICR model by identifying point mutations with whole-genome analysis using next generation sequencing. Determining the relationships between changes in gene expression profiles and point mutation analysis could potentially elucidate the mechanisms of cataract development in the ICR model.

## Results

### Lens opacity progression in ICR rats

**Figure 1:**
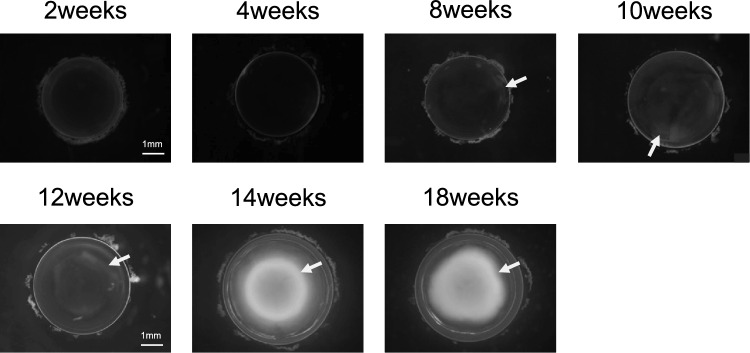
ICR develops opacity in an age-dependent manner. Microscope pictures of lenses just after extraction from 2-, 4-, 8-, 10-, 12-, 14-, and 18-week-old ICR rats. Pictures are shown in grayscale. White arrows indicate lens opacity.

To identify gene expression profile changes occurring concomitantly with progression of ICR lens opacity, we first examined the timing of lens opacity formation. The progression of lens opacity was characterized by extracting and photographing lenses isolated from 2-, 4-, 8-, 10-, 12-, 14- and 18-week-old ICR rats (Fig. 1). Opacities were not present in 2- and 4-week-old ICR lenses, but cortical opacity was detected from the equator of the eye to the cortex in 8- and 10-week-old ICR lenses. In 12-week-old ICR lenses, opacity was detected on the posterior capsule. Moreover, nuclear opacity was detected in 14- and 18-week-old ICR lenses. A prior study reported that opacity at the equator cortex develops by 7 weeks of age, opacity in the posterior capsule cortex develops by 10 weeks of age, and nuclear opacity develops after 3 months of age^17^, consistent with our findings.

### Temporal gene expression profile changes in ICR lenses

**Figure 2:**
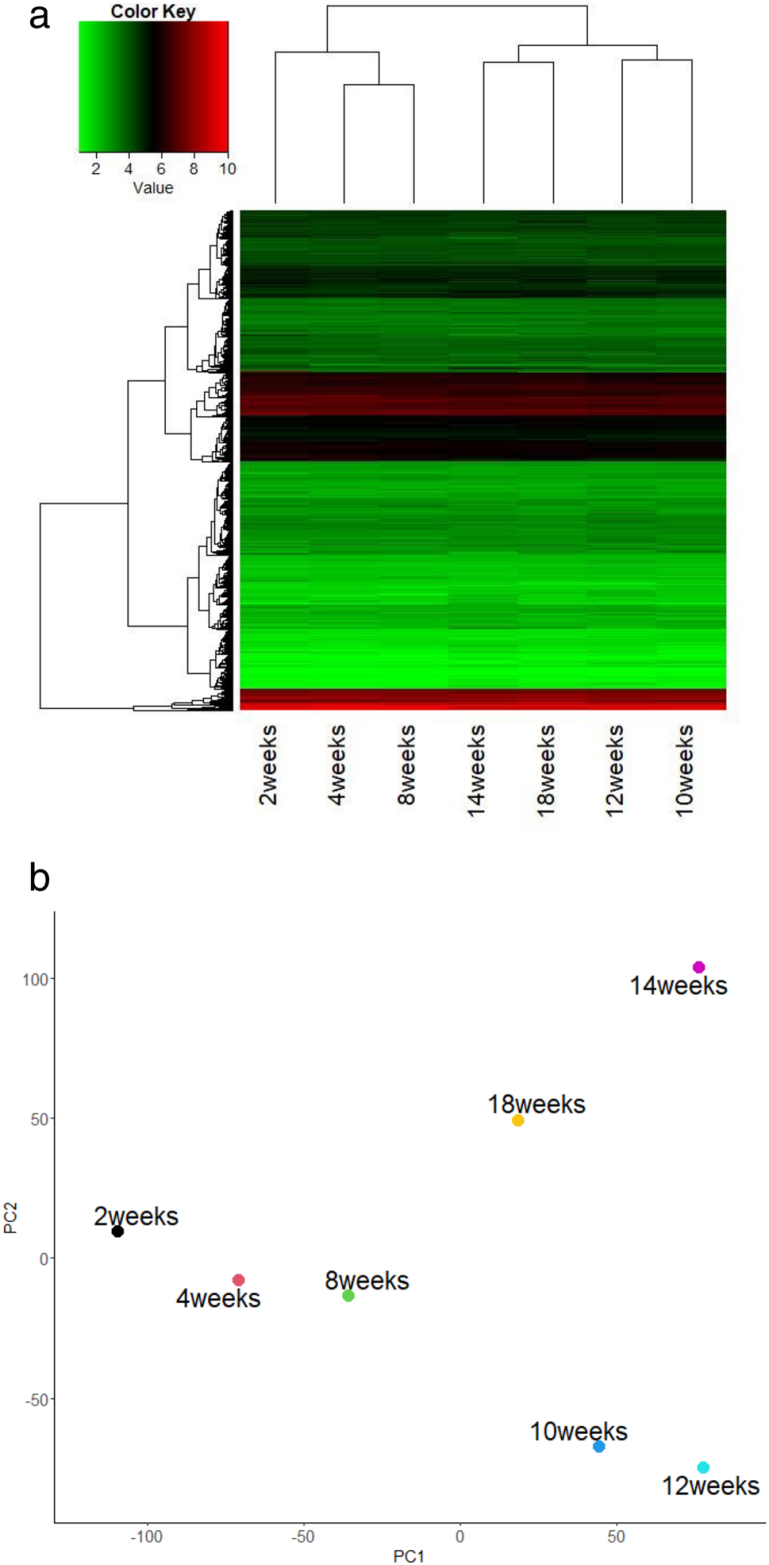
Age-dependent gene expression profiles in ICR lenses. (**a**) Heatmap of expression levels of all genes at each age. Signal values, in order of increasing intensity, are indicated by green, black, and red. (**b**) PCA plot comparing expression levels of all genes at each age.

To examine changes of gene expression profiles occurring over the progression of ICR lens opacity, we conducted microarray analysis of 2-, 4-, 8-, 10-, 12-, 14- and 18-week-old ICR lenses (Fig. 1). A heat map and a PCA plot were generated to analyze the gene expression profiles of microarray (Fig. 2a, b). Heat map analysis revealed the differences of gene expression profiles between 2-, 4- and 8-week-old and 10-,12-,14- and 18-week-old ICR lenses (Fig. 2a). At 10 and 12 weeks of age, the period in which posterior capsule opacification begins, the gene expression profiles were similar between 10- and 12-week-old samples. Also, at 14 and 18 weeks of age, when nuclear opacity begins, 14- and 18-week gene expression profiles were similar.

Next, we conducted PCA plots of all lenses from 2- to 18-week-old lenses demonstrating similar locations for 2- and 4-week-old lenses, in which no opacities are present (Fig. 2b). The other time points at which lens opacity develops were plotted at separate locations. Based on these findings, we used the 4-week-old sample with no cortical opacity as the control, and examined the gene expression changes in 8- and 10-week-old lenses, in which cortical opacities have developed, to identify genes potentially involved in cortical opacity formation.

### Identification of genes involved in lens opacity using microarray analyses

**Figure 3:**
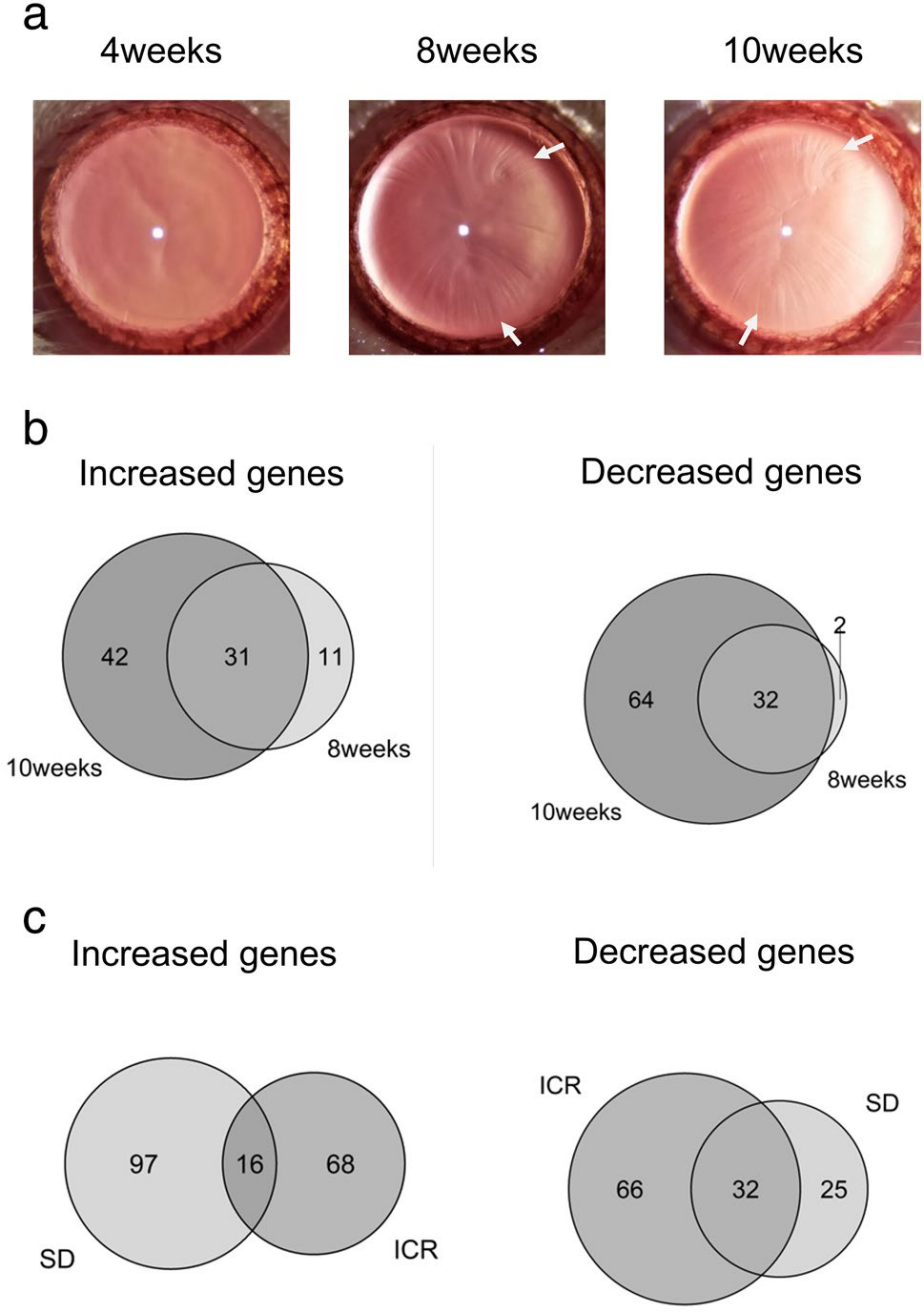
Gene expression profile during cortical opacity formation. (**a**) Pictures of lenses taken by a slit lamp in 4-, 8-, and 10-week-old ICR animals. White arrows indicate lens opacity. (**b**) Venn diagrams illustrating differentially expressed genes from 4- to 8-week-old ICR lenses (8 weeks) and 4- to 10-week-old ICR lenses (10 weeks). Left, more than 1.5-fold increased genes; right, more than 2.25-fold decreased genes. (**c**) Venn diagrams showing expression altered genes from total of 4- to 8-week-old and 4- to 10-week-old in ICR and SD rat lenses. Left, more than 1.5-fold increased genes; right, more than 2.25-fold decreased genes. ICR gene set and SD rat gene set includes differentially expressed genes in both 8-week-old and 10-week-old animals. ICR indicates total of expression altered 4- to 8-week-old and at 4- to 10-week-old in ICR lenses. SD indicates total of expression altered 4- to 8-week-old and at 4- to 10-week-old in SD rat lenses.

To identify genes differentially expressed during formation of cortical opacities, we conducted microarray analysis by extracting three samples each of 4-, 8- and 10-week-old ICR lenses. Slit lamp examination revealed cortical opacity in 8- and 10-week-old ICR rats, while opacities were not present in 4-week-old animals (Fig. 3a).

Subsequently, we conducted microarray analyses of ICR lenses. First, we normalized signal values of the 4-, 8- and 10-week-old samples by average value (n = 3). Subsequently, we identified genes differentially expressed in 4- to 8-week-old or 10-week-old samples. 4- to 8-week-old, there were 42 genes, and 4- to 10 week old, there were 73 genes, for total of 84 genes were increased > 1.5-fold (Fig. 3b, Supplementary Data S1). 4- to 8-week-old, there were 34 genes, and 4- to 10 week old, there were 96 genes, for total of 98 genes were decreased > 2.25-fold (Fig. 3b, Supplementary Data S2).

Subsequently, we conducted microarray analyses of age-matched Sprague Dawley (SD) rats to exclude genes differentially expressed between time points in wild-type animals. Microarray analysis of SD rat lens samples compared with age-matched ICR lens at 4-, 8- and 10-week-old sample (n = 1). 4- to 8-week-old, there were 58 genes, and 4- to 10 week old, there were 67 genes, for total of 113 genes were increased > 1.5-fold (Supplementary Data S3). 4- to 8-week-old, there were 37 genes, and 4- to 10 week old, there were 48 genes, for total of 57 genes were decreased > 2.25-fold (Supplementary Data S4). Moreover, genes that were differentially expressed total of 4- to 8-week-old and 4- to 10-week-old genes in ICR or SD rat used and excluded from the genes in common with SD rat results using a Venn diagram (Fig. 3c). Finally, ICR only 68 genes were increased in ICR rats relative to age-matched wild-type rats, and 66 genes were decreased (Fig. 3c, Supplementary Data S5, S6).

### Quantitative analysis of differentially expressed genes by RT-qPCR

We conducted RT-qPCR to quantify expression levels of differentially expressed genes identified by microarray analysis. First, we excluded genes with unknown functions and genes not able to create primers, for a final total of 53 increased genes and 59 decreased genes. Therefore, we measured expression levels of differentially expressed genes in 4-, 8-, and 10-week-old ICR lens samples (n = 4/time point). 46 significantly (*P* < 0.05) increased genes and 35 significantly (*P* < 0.05) decreased genes (Supplementary Table S1) were identified in 8- or 10-week-old ICR lenses. 24 decreased genes were highly variable between samples in biological replicates of the same time points, and therefore their expression levels did not significantly (*P* < 0.05) differ. Thus, we focused on expression levels significantly (*P* < 0.05) increased genes this study.

**Figure 4:**
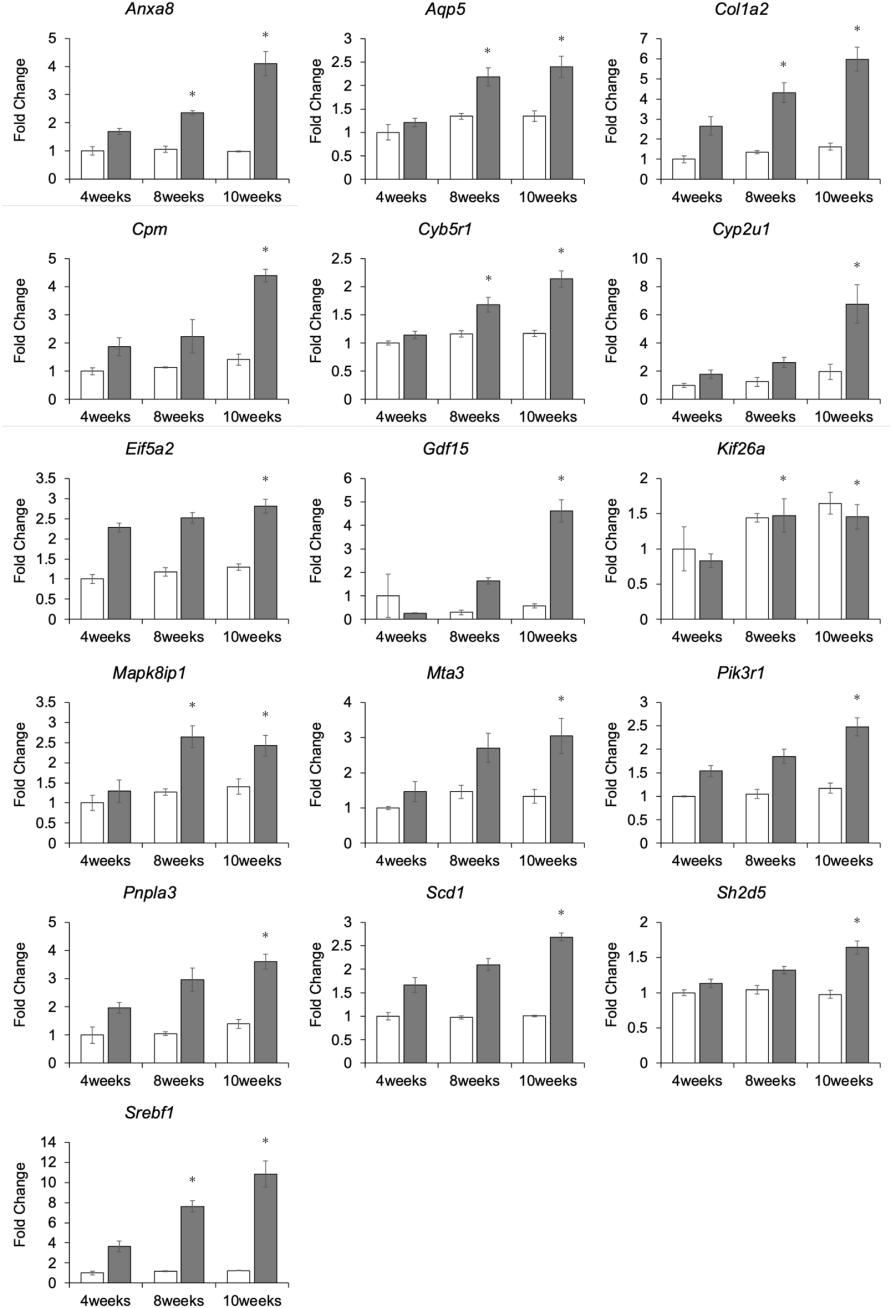
Quantitative RT-qPCR analysis. 16 genes increased expression in ICR only were detected by RT-qPCR among increased genes detected by microarray analysis. White bars indicate SD samples data. Gray bars indicate ICR samples data. mRNA levels were normalized to *Gapdh* mRNA level. Data are presented as mean ± SEM. *P < 0.05 relative to 4-week-old ICR lenses of the same animal.

In addition, RT-qPCR was performed in 4-, 8-, and 10-week-old SD rat lens samples (n = 3/time point) for genes whose expression levels were significantly (*P* < 0.05) increased in ICR to investigate genes involved in lens maturation. If the expression level was significantly (*P* < 0.05) increased in both ICR and SD rat lenses at 4- to 8-week-old or 4- to 10-week-old, we considered the genes to be involved in lens maturation, and if the expression level was significantly (*P* < 0.05) increased in ICR lenses only at 4- to 8-week-old or 4- to 10-week-old, we considered the genes to be important for cortical cataract. As a result, we were able to identify 16 genes whose expression levels were significantly (*P* < 0.05) increased in the ICR only and 30 genes whose expression levels were significantly (*P* < 0.05) increased in both the ICR and SD rat (Fig. 4. Supplementary Figure S1). In subsequent analyses, we focused on the above 16 genes.

### Function analysis of increased genes expression

**Figure 5:**
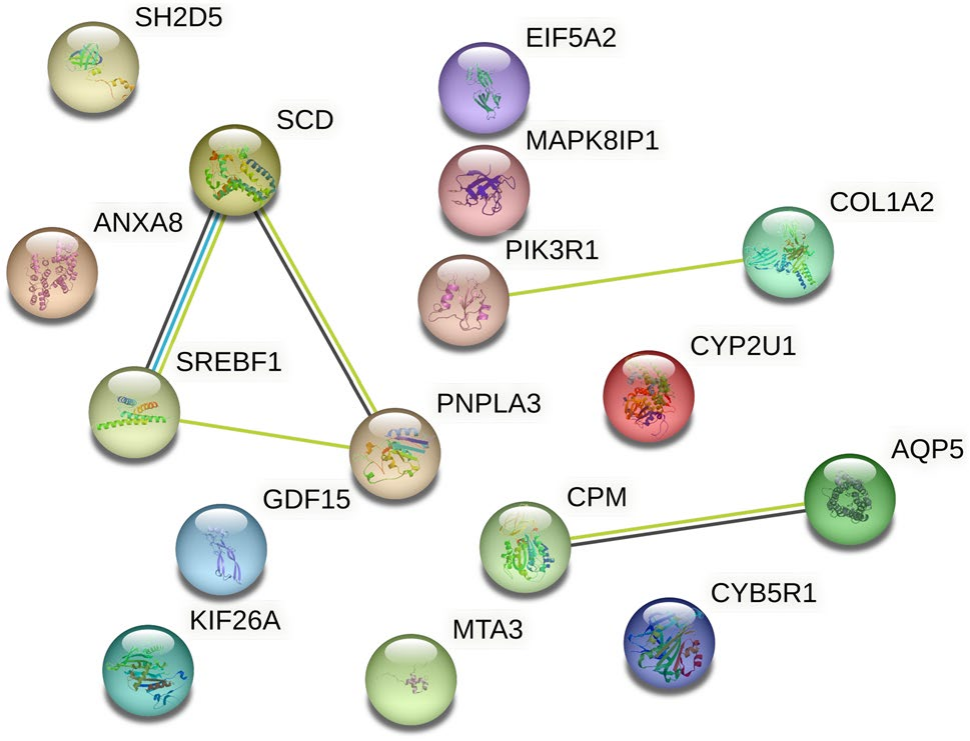
STRING protein interaction analysis. STRING analysis was conducted on the 16 genes detected by RT-qPCR as significantly (*P* < 0.05) increased expression ICR samples only (https://string-db.org/). *Homo sapiens* was chosen as the organism. Scd1 was marked as SCD. The color of each edge indicates the type of relationship between proteins: light blue, from curated databases; purple, experimentally determined; green, text mining; black, co-expression; and light purple, protein homology.

Subsequently, we analyzed the interaction between proteins by STRING to examine the functions of the 16 genes that were significantly (*P* < 0.05) increased gene expression according to RT-qPCR (Fig. 5). Increased genes could be classified into three connections: (i) Epithelial to mesenchymal transition (EMT): *Col1a2,* and *Pik3r1;* (ii) Lens homeostasis: *Aqp5, and Cpm;* and (iii) Lipid metabolism: *Scd1, Srebf1,* and *Pnpla3.* Connection (i) genes were related to EMT^34,35^. Furthermore, *Anxa8* is known to promote EMT in cancer cells^36^. Prior studies have identified that EMT is associated with galactose-induced cataract^37,38^. Connection (ii), especially *Aqp5* transports water through the cell membrane^39^. *Aqp5* increase suggested that water inflow into the lens through cell membranes could be increased. Since the ICR increases the water content in the lens as the cataract progresses, the influx of water into the lens may contribute to the formation of the cataract^17^. *Scd1*, *Srebf1*, and *Pnpla3* in connection (iii) are related to lipid metabolism. *Srebf1* is a transcription factor that regulates of fatty acid synthesis^40–42^; *Scd1* and *Pnpla3* are in fact *Srebf1* target genes. Abnormalities in cholesterol metabolism have been implicated in cataracts^43^. Furthermore, RNA-seq results using mouse lenses in which *Lss*, which catalyzes cholesterol biosynthesis, was conducted knockout and cataract developed, showed decreased expression of cholesterol synthesis pathway genes and increased expression of *Srebf1* and *Scd1* compared to non-cataract developed lenses^44^. Thus, the abnormalities in lipid metabolism that these genes cause, which probably affect cortical cataract formation.

### Next generation sequencing point mutation analysis

**Figure 6:**
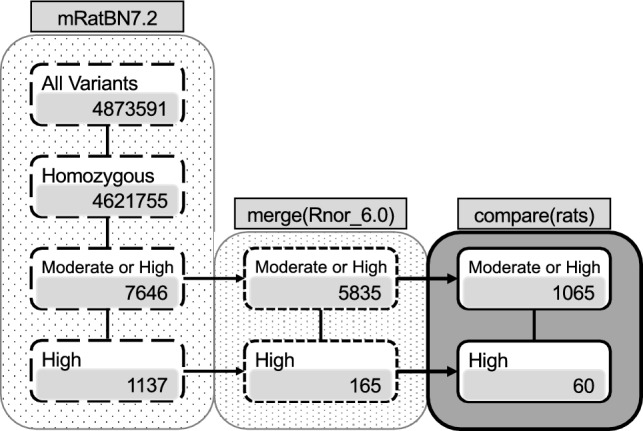
Point mutation analysis and candidate causative mutations. The left column shows the number point mutations identified in ICR animals using mRatBN7.2 as a reference sequence. This detected 4,873,591 point mutations, with 4,621,755 homozygous mutations. Of the selected mutations, 7646 mutations were predicted to be moderate, and 1137 mutations were predicted to be severe according to annotation impact. The center column shows the value of common mutations to the mutations observed by the mutation point analysis of ICR animals using Rnor_6.0 as a reference sequence. Mutations were selected by comparison between mRatBN7.2 and Rnor_6.0. Of these mutations, 5835 were predicted to be moderate by annotation impact and 165 were predicted to be severe. The right column shows the value of eliminated mutations that were identified using the 10 species of rats from the NCBI SRA (Sequence Read Archive) (https://www.ncbi.nlm.nih.gov/sra) compared with mRatBN7.2 as a reference sequence. Finally, 1065 mutations with moderate annotation impact and 60 mutations with high annotation impact were selected.

Next, we determined the whole-genome sequence with the NGS (Next Generation Sequencer) to identify mutations that could cause ICR rats to develop cataracts. We performed an alignment with mRatBN7.2 (NCBI) as a reference sequence. This identified 4,873,591 mutation points on the ICR autosomes (Fig. 6). Because the F_1_ offspring from crossbreeding ICR rats with wild-type rats do not develop cataract, the causative mutation of cataract formation was expected to be homozygous. We identified 4,621,755 mutation points that were present on both chromosomes (Fig. 6). We hypothesized that amino acid-level mutations after translation are also important to cataract formation, so thus selected mutations with moderate anticipated impact, for example, missense mutations and insertions or deletions of one amino acid, and mutations predicted to severely alter gene functions, for example, frameshifts or insertion/deletion of stop codons. The annotation impact, or effect of the mutations, was determined based on sequence ontology. We identified 7,646 locations with moderate mutations, and 1137 locations with severe mutations (Fig. 6).

Next, we analyzed similarly to the above using Rnor_6.0, the other NCBI reference sequence. Similar to above, we identified point mutations by aligning the ICR genome sequence to that of Rnor_6.0. We selected common mutations detected by alignment of the ICR genome sequence with both mRatBN7.2 and Rnor_6.0, as mutations identified from both references were most likely to be impactful for cataract. We identified 5835 common loci with moderate mutations and 165 common loci with severe mutations (Fig. 6).

We compared the ICR sequence with rats that did not develop cataracts from the NCBI SRA (Sequence Read Archive) to identify putative ICR-specific mutations. Ten sequences met these criteria: DRR002169 (F344/stm); ERR185960 (ACI/N); ERR185961 (BN/SsN); ERR185962 (BUF/N); ERR185963 (F344/N); ERR185964 (M520/N); ERR185965 (MR/N); ERR185966 (WKY/N); ERR185967 (WN/N); ERR185968 (LE/Stm). These sequences were aligned with mRatBN7.2 as a reference sequence, and mutations detected in both mRatBN7.2 and ICR were excluded from potential cataract-causing mutations. Finally, 1065 loci were identified as moderate mutations using annotation impact, and 60 loci identified as severe mutations (Fig. 6). The 1065 moderate mutation loci are shown in Supplementary Data S7, and the 60 severe mutation loci are shown in Supplementary Data S8.

Prior studies using the IER model, which is similar to the ICR model, reported that two recessive genes on two loci cause cataract in the IER model^18^. Mutations of genes on the *Cati1* locus of chromosome 8 were identified as being important for cataract formation. *Cati1* is located from *D8Rat68* (18,984,168) to *D8N136* (84,531,276), the SSLP site *D8Rat80* (chr8-47,353,202) and *D8Rat43* (chr8-48,630,131) as center. We analyzed the above 12 rats that did not develop cataract, narrowing the candidate mutations to 11 mutation points in the *Cati1* locus out of 1065 ICR-specific mutations. The identified mutations were predicted to have moderate impact. In these mutation points, we focused to the range from *Thy1* (SSLP) (chr8-44,393,886) to *Cyp1a1* (SSLP) (chr8-58,098,974), which has a high possibility as a location for cataract-causing point mutations, and missense mutation of *Apoc3* (chr8-46,532,966-C > T) and disruptive inframe deletion of *RGD1305464* (function unknown; chr-8–57,650,592-GCAGGGA > G) were selected. APOC3 is an apolipoprotein, and *Apoc3* mutations potentially dysregulate metabolism of lipids and cholesterol, which is likely to contribute to cataract formation^43,45^.

Also, mutations of genes of the *Cati2* locus on chromosome 15 detected in IER are concerned with a cataract developing period^18^. *Cati2* is located from *D15Rat52* to *D15Rat20*. Since the position of D15Rat52 in mRatBN7.2 was unreleased, the number of bases between *D15Rat52* and *D15Rat20* in Rnor_6.0 was calculated and the position of *D15Rat52* in mRatBN7.2 was estimated. As a result, *Cati2* was estimated to be located at *D15Rat52* (32,677,550) to *D15Rat20* (52,813,021). We performed the same analysis as for *Cati1* to narrow down the mutation points. As result, mutations in 4 genes were identified, stop lost of *Phf11b* (chr15-33,378,050-T > G), splice donor variant & splice region variant & intron variant of *Ppk* (chr15-40,027,471-GGTGAGTGAGTGA > G), missense mutation of *Nkx2-6* (chr15-44,446,376-G > C), conservative inframe insertion of *Sucla2* (chr15-48,760,331-T > TGCA). However, since mutations in *Cati2* only do not cause cataracts, mutations in *Cati1* together cause cataracts, *Cati2* may have a similar function to the *Cati1* gene. In the SCR nuclear cataract model, a missense mutation of *Fdft1*, which catalyzes the conversion of farnesyl pyrophosphate to squalene, was identified^13^. Interestingly, the same missense mutation in the SCR model occurs in the ICR model, in which a common mutation point was detected at *Fdft1* (chr15-37,423,413-A > T) on chromosome 15. However, the *Fdft1* was not selected as a specific mutation of the ICR model as a result of aligning the ICR sequence to sequences of 12 rats that do not develop cataract. *Lss* and *Fdft1* mutations have been reported in the SCR model^13^, and mutation of *LSS* causes cataract in humans^46^. Therefore, abnormal cholesterol metabolism can cause cataracts. *Fdft1* only mutated in rats that do not develop cataracts, but functions two steps upstream of *Lss* in the cholesterol biosynthesis pathway^13^, and the mutation further inhibits cholesterol biosynthesis, suggesting that *Fdft1* plays an assistive role in cataract development. Hence, cataract development in the ICR model could be due to abnormal cholesterol metabolism caused by the mutations of *Apoc3* and *Fdft1*.

## Discussion

Induced and heritable cataracts are used as cataract animal models. Because humans develop cataract with aging, genetic models with aging-dependent cataract development are likely to be more relevant to human cataract studies. Depending on opacity location, cataract is classified to nuclear, cortical, or posterior subcapsular cataract. In this study, we focused on cortical cataracts, which develop at a young age. ICR is a well-known as a rat cortical cataract model, but most studies of ICR model have been concerned with protein changes, and few studies have evaluated changes gene expression changes^26,27,30–32^. Therefore, we sought to identify the mechanism of cataract development in the ICR model using microarray and point mutation analyses. Microarray studies compared gene expression in ICR lenses before and after the time point for development of cortical cataract.

RT-qPCR results suggest that genes whose expression increased from 4-week-old, when cataracts do not develop, to 8- or 10-week-old, when cataracts do develop, are important in the development of cataracts. In addition, there were genes whose expression increased from 8- to 10-week-old (Fig. 4). These genes were also important in the progression of cataracts, as their expression increased with the progression of cortical cataracts.

Functional analyses of expression changes in selected gene groups suggested that EMT, inflow of water into the lens and abnormal lipid metabolism are associated with cataract in the ICR model (Fig. 5). EMT in LECs is an abnormal form of transcriptional programming that causes a phenotype of increased invasiveness and abnormal activation of cell proliferation^47^. EMT contributes to development of posterior subcapsular cataracts observed frequently after cataract surgery^48^. *Col1a2* encodes the 2α(I) chain of type-I collagen as fiber collagen^49^, and TGF-β2 induction of LEC EMT was less robust in *COL1A2* knockdown conditions^50^. Prior studies reported the involvement of EMT in galactose-induced cataract^37,38^, although no common differentially expressed genes were identified between the galactose-induced cataract model and the ICR model. It is thus possible that EMT in diabetic and ICR cataracts occurs via distinct mechanisms.

In ICR lenses, water content increases with the progress of opacity^17^. LEC expression of *AQP1* and *AQP5* are increased in cataract patients^51^. Similarly, we detected significant (*P* < 0.05) increase of *Aqp5* before and after onset of cortical cataract, and *Aqp1* was non-significantly increased. This suggested that increased lens water content could be due to increase of *Aqp5,* which would increase water permeability of the cell membranes.

Lipid metabolism-related genes were also increase during cataract formation. This suggests that lipid metabolism, including dysregulation of cholesterol and fatty acids, could contribute to cataract. Deletion and inhibition of cholesterol synthesis enzymes causes cataract formation^43^. Dysregulation of lipid metabolism could potentially increase levels of lipid peroxides generated by oxidation of polyunsaturated fatty acids^52^. Transcriptomics studies of the mutant mouse cataract model of *Lss*, revealed robust increases in the expression levels of genes related to fatty acid metabolism, such as *Srebf1* and *Scd1,* and decreased expression of *Srebf1* and *Scd1* involved in the cholesterol synthesis pathway^44^. In the ICR model, we also detected increase of genes related to fatty acid metabolism, suggesting that increased lens fatty acids could contribute to cataract development.

NGS mutation point analysis identified 1,065 point mutations anticipated to have moderate impacts on transcript products and 60 point mutations predicted to severely impact transcript products. In this analysis, we focused on the locus reported in the IER, but discussion of previous paper it was mentioned that it would be necessary to consider the existence of minor genes in addition to *Cati1* and *Cati2* and genes related to cataracts other than *Cati1* and *Cati2* were mentioned in the discussion^18^. Therefore, we investigated genes other than *Cati1* and *Cati2* that are involved in cataracts. A missense mutation of *Gja8* (chr2-184,491,323-C > A) was detected. *Gja8* encodes the gap junction protein Cx50, and is expressed widely from LECs to fiber cells^53^. Prior reports identified that *Gja8* mutations are related to cataract formation in humans and rodents^54,55^. Interestingly, another mutation in *Gja8* (R340W) occurs in UPL rats, which is predicted to result in a non-conservative amino acid change at the carboxyl terminus of Cx50^15^. The mutation we found is predicted to result in the replacement of valine by leucine (V353L), mutation at the carboxyl terminus. The carboxyl terminus contains phosphorylation sites for various kinases and is thought to be important for Cx50 specific function ^56^. Furthermore, the circulation of water and ions through gap junctions is important for lens growth and maintenance of transparency^55,57^. Thus, Mutations in *Gja8* (V353L) may cause disruption of lens homeostasis and are implicated in cataracts in ICR models. Moreover, SREBF2 is a transcription factor that regulates cholesterol synthesis, and we detected a splice donor variant, a 3_prime_UTR_variant, and an intron_variant of *Srebf2* (chr7-113,719,718-TTTTTG > T). This mutant is thought to cause loss of function of SREBF2 which is encoded by *Srebf2*. The lens is rich in cholesterol and the supply of cholesterol is dependent on de novo synthesis^58^. Cholesterol deficiency has been reported to cause cataracts in the lens^43^, and several animal models with defects in cholesterol biosynthesis genes such as *Srebf2*, *Lss* and *Fdft1* show a cataractous phenotype^13,59^. Mutations in *Srebf2* may therefore also cause cataracts in ICR.

Furthermore, in the IER model, which is closely related to the ICR model, mutations of genes on the *Cati1* locus on chromosome 8 contribute to cataract formation^18^. In this study, causative mutations in the *Cati1* locus in ICR rats were narrowed down to *Apoc3* (chr8-46,532,966-C > T) and *RGD1305464* (function unknown, chr8-57,650,592-GCAGGGA > G). APOC3 is a lipoprotein that inhibits lipoprotein lipase, which breaks down triglycerides^45^. Accordingly, circulating triglycerides and APOC3 protein are decreased in *APOC3* mutants^60^. Moreover, a meta-analysis identified that statins that decrease plasma low-density lipoprotein cholesterol also decrease circulating APOC3, suggesting potential involvement of APOC3 in cholesterol metabolism^61^. Therefore, *Apoc3* missense mutation probably disrupt lipid and cholesterol metabolism.

Transcriptomic and point mutation analyses suggested that abnormal lipid metabolism is involved in cataract formation in the ICR model. Because lens cholesterol is synthesized *de novo*^58^, lens cholesterol could be decreased by the mutations of *Srebf2*, *Apoc3,* and *Fdft1*. Cholesterol functions as an antioxidant in the lens, presenting the possibility that decreased lens cholesterol could cause oxidative stress^62^. Lens lipid peroxides are increased in ICR animals before and after cataract development^63^. However, microarray analysis did not identify differential expression of genes related to the oxidative stress response, underscoring the need for further investigation. Moreover, SREBF1, a transcription factor that regulates lipid synthesis, promotes EMT in breast cancer cells^64^. Therefore, EMT in the ICR lens could be due to abnormal lipid metabolism.

## Materials and methods

### Animals

ICR/Ihr rats were supplied by the National BioResource Project—Rat, Kyoto University (Kyoto, Japan). We used breeding individuals for experiments. SD rats were purchased from Sankyo Laboratory Service. ICR and SD rats used male lenses in Microarray analysis and male and female lenses in RT-qPCR analysis. Rats were euthanized with CO_2_ asphyxiation as described previously^65^. All experiments were approved by the Animal Research Committee of the University of Fukui (Approval number: 28091) and conducted in accordance with the University of Fukui regulations on animal experiments and the Association for Research in Vision and Ophthalmology Statement for the Use of Animals in Ophthalmic and Vision Research. The study was reported in accordance with ARRIVE guidelines.

### Slit lamp observation

Mydriatics were applied to ICR eyes. Five minutes later, lenses were observed and photographed with a LUMIX GF10 digital camera (Panasonic)-equipped portable slit lamp (Kowa).

### Microscopy

Lens images were taken in a dark room using an SZX12 stereomicroscope fitted with a DP58 camera (Olympus), as described previously^65^. Photographs were captured in a 35 mm Petri dish containing 7 mL PBS.

### Microarray data analysis

Lenses were extracted from the specified ages of ICR rats and subjected to microarray analyses. A GeneChip Rat Gene 2.0 ST array chip (Thermo Fisher Scientific) was used to perform microarray experiments as described previously^65^. Preprocessing and data analyses were performed using R software. First, all data were normalized with the Robust Multi-array Average algorithm, and probes with no correspondence with genes were excluded. In analysis of expression level change, genes with signal value < 5 in all samples were excluded. The signal values of 4-, 8-, and 10-week-old samples were normalized to the mean value (n = 3). Selected genes were functionally evaluated using STRING (https://string-db.org/)^66^.

### RNA extraction, cDNA preparation, and real-time RT-qPCR

We performed an RNA extraction of the lens and real-time RT-qPCR as described Kanada et al.^65^. Primer sequences are specified in Supplementary Table S2. Gene expression levels were normalized to *Gapdh*. To assess differences between 4-week-old samples and 8- or 10-week-old samples, homoscedastic approval was conducted as described by Nagaya et al.^37^.

### Next generation sequencing analysis

Genomic DNA was extracted using NucleoSpin DNA RapidLyse (Takara Bio). TruSeq DNA PCR-Free Library Prep kits (Illumina) were used for library regulation. Whole-genome sequencing was performed with a NovaSeq6000 system (Illumina) under 150-bp paired-end reads.

### Mutation point analysis

The DNA libraries of 12 species of rats that do not develop cataracts were downloaded from the NCBI SRA (Sequence Read Archive) (https://www.ncbi.nlm.nih.gov/sra). Subsequently, data were compared to ICR DNA sequence on a Linux environment to select mutation points.

### Statistical analysis

To assess the differences 4- to 8-week-old, 4- to 10-week-old ICR lens samples (n = 4) and 4- to 8-week-old, 4- to 10-week-old SD rat lens samples (n = 3) by RT-qPCR, the two-tailed Student’s t-test was performed if homoscedasticity was observed, and a two-tailed Welch’s t-test was performed if homoscedasticity was not observed. *P* < 0.05 was considered statistically significant. Statistical analyses were conducted with Microsoft Excel.

### Supplementary Information

Supplementary Information 1.

Supplementary Information 2.

Supplementary Information 3.

Supplementary Information 4.

Supplementary Information 5.

Supplementary Information 6.

Supplementary Information 7.

Supplementary Information 8.

Supplementary Information 9.

## Data Availability

Microarray data are available in the GEO repository under the accession number GSE230323 (https://www.ncbi.nlm.nih.gov/geo/query/acc.cgi?acc=GSE230323). Nucleotide sequence data reported are available in the DDBJ Sequenced Read Archive under the accession numbers DRR409762 (https://ddbj.nig.ac.jp/resource/sra-run/DRR409762).
