# Supplementary material for: Gene profiles and mutations in the development of cataracts in the ICR rat model of hereditary cataracts
Source: Sci Rep. 2023 Oct 24;13:18161. doi: 10.1038/s41598-023-45088-1 (PMC10598066; doi:10.1038/s41598-023-45088-1)
Supplement: Supplementary file 7 — Supplementary Information 7. [file 41598_2023_45088_MOESM7_ESM.docx]

Gene profiles and mutations in the development of cataracts in the ICR rat model of hereditary cataracts

Masaru Takashima^1^, Kei Taniguchi^1^, Masaya Nagaya^1^, Shunki Yamamura^1^, Yoshihiro Takamura^2^, Masaru Inatani^2^ and Masaya Oki^1,3,*^

^1^Department of Industrial Creation Engineering, Graduate School of Engineering, University of Fukui, Fukui, Japan;

^2^Department of Ophthalmology, Faculty of Medical Sciences, University of Fukui, Fukui, Japan;

^3^Life Science Innovation Center, University of Fukui, Fukui, Japan

Correspondence and requests for materials should be addressed to M.O.(email: ma4sa6ya@u-fukui.ac.jp)

**Supplementary Figure S1: RT-qPCR results with increased expression in ICR and SD rats**

30 genes increased expression in ICR and SD rat were detected by RT-qPCR among increased genes detected by microarray analysis. White bars indicate SD samples data. Gray bars indicate ICR samples data. mRNA levels were normalized to *Gapdh* mRNA level. Data are presented as mean ± SEM. * P < 0.05 relative to 4-week-old lenses of the same animal.

**Supplementary Table S1. List of 81 genes that were significantly (P<0.05) increased or decreased from 4- to 8-week-old or 10-week-old ICR samples by RT-qPCR.** The number of genes with increased expression is 46, and the number of genes with decreased expression is 35.

| **Increased genes** | | **Decreased genes** | |
| --- | --- | --- | --- |
| *Abhd6* | *Kif26a* | *Aif1l* | *Pnkd* |
| *Anxa1* | *Lgsn* | *Aplp1* | *Rnf180* |
| *Anxa8* | *Lims1* | *Caprin2* | *Slc20a2* |
| *Aqp5* | *Mapk8ip1* | *Cbln2* | *Sorbs2* |
| *Arl15* | *Mta3* | *Cpt2* | *Spire1* |
| *Cald1* | *Nfix* | *Dnase2b* | *Tmem100* |
| *Capn6* | *Ntn4* | *Eif4e* | *Tmem154* |
| *Cd44* | *Osgin2* | *Fbxo7* | *Tnnt2* |
| *Col1a2* | *Pik3r1* | *Fxyd6* | *Tnrc6b* |
| *Cpm* | *Pnpla3* | *Gca* | *Trex1* |
| *Cyb5r1* | *Ramp2* | *Ggct* | *Ube2o* |
| *Cyp26a1* | *Rcan2* | *Gje1* | *Whamm* |
| *Cyp2u1* | *Scd1* | *Hmox1* |  |
| *Dapl1* | *Sh2d5* | *Idi1* |  |
| *Dtnbp1* | *Slc12a2* | *Igf2bp2* |  |
| *Eif5a2* | *Slc25a28* | *Limd1* |  |
| *Emp2* | *Slc6a3* | *Loxl1* |  |
| *Erich5* | *Srebf1* | *Metap1* |  |
| *Fn3k* | *Stk35* | *Mtch2* |  |
| *Gdf15* | *Trpm1* | *Myo18a* |  |
| *Gltscr2* | *Vwa5a* | *Nhsl1* |  |
| *Hnrnpul2* | *Zbtb4* | *Nlgn1* |  |
| *Hopx* | *Zcchc24* | *Nt5dc2* |  |

**Supplementary Table S2. List of used for real-time RT-qPCR.**

| **Increased genes** | | |
| --- | --- | --- |
| **Gene** | **Forward primer** | **Reverse primer** |
| *Gapdh* | 5'-GAGACAGCCGCATCTTCTTGT-3' | 5'-CGACCTTCACCATCTTGTCTATGA-3' |
| *Abhd6* | 5'-GCTCGTACGTCCGCTTCAA-3' | 5'-GCGAACGTCGACAAGACCTT-3' |
| *Anxa1* | 5'-GTGGATGAGGCAACCATCATT-3' | 5'-TGCTGGCGCTGAGCATT-3' |
| *Anxa8* | 5'-CGAGAGCGTCAGGCTTGTC-3' | 5'-TCAGCATGGAGGGAACGAA-3' |
| *Aqp1* | 5'-TTCAGCTCAGGGCTTGCAT-3' | 5'-TCTGGCTTGGTGGGATTGA-3' |
| *Aqp5* | 5'-CACCCTCATCTTCGTCTTCTTTG-3' | 5'-CAGAGCCGAGGGCCACTT-3' |
| *Arl15* | 5'-CGAGGCCATGAGGATGTGA-3' | 5'-CGTGATGGGTTCAAGGCTTT-3' |
| *Cald1* | 5'-CCGCATCAATGAATGGCTAA-3' | 5'-TTGGGAGCGGGTGACTTG-3' |
| *Calr4* | 5'-GCGATGGATGGACAAAACG-3' | 5'-GCCGGAATTTACCGTAATCTGA-3' |
| *Capn6* | 5'-CTCACACACGCGCAGGTAA-3' | 5'-CCTTATTCTTGCCCACTGACTGA-3' |
| *Cd44* | 5'-ACAACCCCCAGACCACTCTTT-3' | 5'-AGTACCAACTGGCTTGCGAAA-3' |
| *Col1a2* | 5'-GCCTTCATGCGCCTGCTA-3' | 5'-TCTTGCAGTGGTAGGTGATGTTCT-3' |
| *Cpm* | 5'-TTCCACAGGAAGTGAGGACACA-3' | 5'-GCCCATATATCCCTTTAGAGTCTCAA-3' |
| *Cyb5r1* | 5'-CCAGTGCTTTCTGCTTTTTGC-3' | 5'-AGGTCCTCCCGCAGGATTAT-3' |
| *Cyp26a1* | 5'-TGCATGATTCCTCGCACAA-3' | 5'-CTCGGTTGAAGGCCTGCAT-3' |
| *Cyp2u1* | 5'-CACAGAGGGACTGCACGTCTT-3' | 5'-GGGACAGGAAGTGCAAAGTCTT-3' |
| *Dapl1* | 5'-TCGTCCCCGCTGAAAGG-3' | 5'-ATTCGCATCCCTCCAGCTT-3' |
| *Dtnbp1* | 5'-GCGTGCAGCAGGATTTCAC-3' | 5'-TGCTTCTTTTGACTTGTCGCTTA-3' |
| *Eif5a2* | 5'-GCCGGAAGTTCCCTCAGAA-3' | 5'-AATCAATTTCGTCTGCCATGGT-3' |
| *Emp2* | 5'-GTGGCTTTCGCCTTCACTTT-3' | 5'-TTGCGCTTCCTCAGGATCAT-3' |
| *Erich5* | 5'-CCAGACGGAAGCCAGCTAGT-3' | 5'-CACCATCGGGAGCTTCACA-3' |
| *Fn3k* | 5'-TGAGCAGGGCCCCATTATT-3' | 5'-TCAAACTCAGAATGGCCGTAGA-3' |
| *Gdf15* | 5'-CCAGCTGTCCGGATACTCAG-3' | 5'-GGTAGGCTTCGGGGAGACC-3' |
| *Gltscr2* | 5'-TGGAGAAGCGGGCTTTCC-3' | 5'-GGCAGAGGCTCCAACATCTG-3' |
| *Gpd1* | 5'-TCGCTCGCACCGGAAA-3' | 5'-TGCCCGTTCAGCATCTCTTT-3' |
| *Hist1h2ao* | 5'-GCAAAGGCGGCGCTAAG-3' | 5'-TAGTGATGCCCTGGATGTTGTC-3' |
| *Hnrnpul2* | 5'-CTGCTGCCCCCCTCTGA-3' | 5'-GGTTTCTCTTGTTGCGGTTGTT-3' |
| *Hopx* | 5'-CTTGACCGCCACTTTCCAAT-3' | 5'-GCAGCTCTGGAATGCTCTGAA-3' |
| *Kif26a* | 5'-CGAGCCGGCAAAATGG-3' | 5'-TCACACCGTTCTAGGGACATGT-3' |
| *Lgsn* | 5'-GCGGCTGAATCCGATGAG-3' | 5'-TCAAAGGGATCTCGGAAGGTT-3' |
| *Lims1* | 5'-GAGCAACATGGCCAATGCT-3' | 5'-TCAAAGCCCCCCTTGCA-3' |
| *Mapk8ip1* | 5'-CACCTCCGAAGGACTTTCCA-3' | 5'-GATGAGAACAGGGTCCTCACTGT-3' |
| *Mta3* | 5'-TGCAAGGCATCCTTTTTTGC-3' | 5'-TCCCACATCCCCAGTTCATC-3' |
| *Nfix* | 5'-GAGGAGCGCGCAGTGAA-3' | 5'-TGCCCACTTCTGCTTGATCTC-3' |
| *Ntn4* | 5'-GCGAGAATGCTGATCTCACTTG-3' | 5'-TGGGCCCCGTTGCA-3' |
| *Osgin2* | 5'-AACTGGGAAATCAGAGGCTATCAG-3' | 5'-CAAAGAGGCAGAAGGGAACGT-3' |
| *Pik3r1* | 5'-GCAAGAGCCCTCTCTGAAATTTT-3' | 5'-AGAGCTGGCTGCTGGGAAT-3' |
| *Pnpla3* | 5'-CCTCTCGATCACATCATGGAGAT-3' | 5'-TTGCGGCTCCTGGCTTT-3' |
| *Pole3* | 5'-TGTTTTCGTTTTGTATGCCACAT-3' | 5'-TCTTGCGCTTTCCTTTCATTG-3' |
| *Rab40c* | 5'-CGGCATGGCATGGAGAAG-3' | 5'-AGGTCCTGCAGGCTGAAAACT-3' |
| *Ramp2* | 5'-GCTGGGCGCTGTCTCAAC-3' | 5'-GCTGTCCTCAGTAGGATGAGATTG-3' |
| *Rcan2* | 5'-GGGTATGCCCCACCTTGTG-3' | 5'-CGTGGATGCCCCTTGTCTAT-3' |
| *Sap30bp* | 5'-GCAGGACTGTGCAACCCAGTA-3' | 5'-GCGTATCAGTCCCAAACAAAGG-3' |
| *Scd1* | 5'-CCGTGGCTTTTTCTTCTCTCA-3' | 5'-TCTTTGACAGCCGGGTGTTT-3' |
| *Sh2d5* | 5'-GCTTGGTGGCTGCTTTAGGA-3' | 5'-CCCAACGGTCAGCTCTGTGT-3' |
| *Slc12a2* | 5'-CAGGTGATGAGCATGGTGTCA-3' | 5'-TGAGAGTGTGGCTGAAAAGATACC-3' |
| *Slc25a28* | 5'-CTGCCCTTCCAGCACATTCT-3' | 5'-CCTTCCTTCCAATTCCTCTTGA-3' |
| *Slc6a3* | 5'-TCCGGGAGAAACTGGCCTAT-3' | 5'-CCCCTCTGTCCACTAGCTGATG-3' |
| *Srebf1* | 5'-TGATGGAGACAGGGAGTTCTCA-3' | 5'-AGCGTCAGAACAGCTATTTAGCAA-3' |
| *Stk35* | 5'-TGGGTTAGCCCAGCGTATG-3' | 5'-GGCGCAGGTAAAGCTGTGA-3' |
| *Trpm1* | 5'-CCGCGGGCAGATTTCA-3' | 5'-CGCCGTGTTCAGACTTGGT-3' |
| *Vwa5a* | 5'-CAGCCTTCAGTCTGGAGTTCTCA-3' | 5'-TGCACTGGCTTGTTAAGCTCTTT-3' |
| *Zbtb4* | 5'-TCAGATCACTGTGCGAATTGG-3' | 5'-CTCCCGGACGTAGGTCAGTTT-3' |
| *Zcchc24* | 5'-GGTCTTTGCAGCCATCTTCTG-3' | 5'-AAGTTGGCCATAAGAGCAAAGG-3' |
| **Decreased genes** | | |
| **Gene** | **Forward primer** | **Reverse primer** |
| *Gapdh* | 5'-GAGACAGCCGCATCTTCTTGT-3' | 5'-CGACCTTCACCATCTTGTCTATGA-3' |
| *Aif1l* | 5'-GCACTCAGCAACAGGTTCCA-3' | 5'-TCCTGCCGGGCTTTGA-3' |
| *Aplp1* | 5'-CTCCTCTGGGAGAGAGAAGCTAAC-3' | 5'-GGGCAGATGCATTCACCTTT-3' |
| *Apoe* | 5'-TCCATTGCCTCCACCACAGT-3' | 5'-GGCGTAGGTGAGGGATGATC-3' |
| *Caprin2* | 5'-TCGGCGTGCAGTTTGTGA-3' | 5'-GGCCCTAACCAAGTGCATTC-3' |
| *Cbln2* | 5'-GCCCGGTAAAGGCTCAGAAC-3' | 5'-TACCAGGCACTTGCCCTCTAG-3' |
| *Cpt2* | 5'-TTCGACACCACGGTGAAAAC-3' | 5'-CCTTGCCACCTCTCTGGAACT-3' |
| *Crygn* | 5'-CGTGGGCACCAGCTACAAGT-3' | 5'-TCGCAGTACAGGCCATGGTA-3' |
| *Dnase2b* | 5'-TCCCCCAGTTCCGGAAA-3' | 5'-GCATATTGCCTCCCCGAGTT-3' |
| *Eif4e* | 5'-CCCAGAACCCACAGATGTGTT-3' | 5'-GTGGTAACTCTACCCGGCTGTT-3' |
| *Enpp2* | 5'-AGGATTCACAGCAAGTCGAATTAA-3' | 5'-GCACTGTGGGAGGTCCTTCA-3' |
| *Enpp6* | 5'-CCTTCCTGCTGCTGTTTGG-3' | 5'-TCCGGTGGGCAGAAGCT-3' |
| *Fbln1* | 5'-CGTCCTGAAGCTGGAGATGAA-3' | 5'-CGTTCCGGTGGGAAACC-3' |
| *Fbxo7* | 5'-TGGTGCATCTCCTTATGCTTGA-3' | 5'-GGATGCTGCTTTGGCTTCA-3' |
| *Fxyd6* | 5'-CAGCGGGAGAGAGCAACAG-3' | 5'-TTTGACTCCCGTAGGCGTTT-3' |
| *Gca* | 5'-GCGCTGACGCCGACTTA-3' | 5'-GCCATGACCAGGAGATCGA-3' |
| *Ggct* | 5'-CGGTATCCCCGCTCTTCTG-3' | 5'-GCTCGCCATGTCCACTTGT-3' |
| *Gje1* | 5'-CGGCTCAGTGCGGATGTT-3' | 5'-CCTCGTTCCCATAGACAGCAA-3' |
| *Hmox1* | 5'-TCGGTAGAGGCGGCTGTTC-3' | 5'-GTCAACATGGACGCCGACTA-3' |
| *Hunk* | 5'-AAACAGCCGTCGCCTTCA-3' | 5'-TCTTTGAGCAGGGCTTTGGT-3' |
| *Idi1* | 5'-CGCTGTGTTCTAGGTCAGATCAGA-3' | 5'-CATCAAGATTGCTGGCATTGA-3' |
| *Igf2bp2* | 5'-CATCCGCGCCATCGA-3' | 5'-CCATGATTTTCCCATGCAACT-3' |
| *Iqgap2* | 5'-TGGGCATCTTTGACGTCAGA-3' | 5'-GAATATTGAGTTGCACCTTTTCCA-3' |
| *Limd1* | 5'-GTAGGCCCCGGACACTGA-3' | 5'-GCCTCAAGGCCCAGATCA-3' |
| *Loxl1* | 5'-CATCCACGGGCAAGAAAGTT-3' | 5'-GTGCTGTCCTCCAGGCAAAA-3' |
| *Metap1* | 5'-TCCAGTGTCCCACCTGCAT-3' | 5'-TCCTGCGAGCAGAAGTACGA-3' |
| *Mfap4* | 5'-GCCGGTCCCTGTCTTCTGT-3' | 5'-TTCTGGAAAACCGTCCACTTG-3' |
| *Mtch2* | 5'-GGGCTCCGGTCTCACCAT-3' | 5'-ACCTGGATGAGCACTTTCACGTA-3' |
| *Myo10* | 5'-GTATGATGTCCGAGGCATCTTG-3' | 5'-GAGCAGGTTGAGAAGGTCATCTC-3' |
| *Myo18a* | 5'-AGCAATTTGCCCGTCATGA-3' | 5'-AGATCGCTGAGGACAGCTCTTC-3' |
| *Nhsl1* | 5'-CCCCCTCTCGTAAAAATTACACA-3' | 5'-AATGTATGCAACGGGCACAAC-3' |
| *Nlgn1* | 5'-TGTGTTGGCAAGCTACGGTAAT-3' | 5'-CCAAGTACCCCAAGCCGATA-3' |
| *Nt5dc2* | 5'-GCATCACGAAGGCCCTGTT-3' | 5'-TTGTGGAAGGTGCGGAAGAT-3' |
| *Pgap1* | 5'-CCCGTGTTTTGTGATCATTCC-3' | 5'-TTGCGCGTCCTACATGAAGTT-3' |
| *Pnkd* | 5'-CCCGGCCCCCAAGA-3' | 5'-CGTGAGCGAAAGGCACAAA-3' |
| *Ptprk* | 5'-CACTTTCAACGTCACCATTTGC-3' | 5'-TCGGCCCTGCTCTCATTG-3' |
| *Reln* | 5'-CAGCTTTCGACTACCCTATTAACCA-3' | 5'-CGTAGTGGCACAGAAGCTATCG-3' |
| *Rnf180* | 5'-TGGAGGCCTCGGACCAA-3' | 5'-CTGGCAGAGCGAAGGAAGTC-3' |
| *Slc13a3* | 5'-CTGGCTGGATTCTGCCTTTT-3' | 5'-ACCTCTAGCTGCCAGGTATCATCT-3' |
| *Slc13a4* | 5'-TGCTGCCTGTGGGCAAT-3' | 5'-TCTGGCAGTGCCCATAGCT-3' |
| *Slc20a2* | 5'-CGTCCAGTGGCTTCACTATTGA-3' | 5'-CCAATGTTGGAGGCGATCAC-3' |
| *Slc4a5* | 5'-CGAAGGTTTGATGGACGTCTAAG-3' | 5'-CGTGCAAACCTGCTCAACAC-3' |
| *Slc6a13* | 5'-CTCCAGCGACACCGATGAC-3' | 5'-CCCTAGCAGTTAGACTCCAGTTCTG-3' |
| *Slc6a9* | 5'-GTACTTCACGGCCACATTTCC-3' | 5'-GGGTCACTCCACGAACAAACA-3' |
| *Slc7a11* | 5'-CCTGGCATTTGGACGCTACAT-3' | 5'-TCAGAATTGCTGTGAGCTTGCA-3' |
| *Sned1* | 5'-TTCCCTGTCTATAGCCTCCCAATA-3' | 5'-CCTCCCCCAGAGAGTCATTTC-3' |
| *Sod3* | 5'-GCGACACGCACTCCAAAGA-3' | 5'-CCGCCTCCCGTTGTTTT-3' |
| *Sorbs2* | 5'-TCCGCCCCTTTGCAGTTA-3' | 5'-TAAGCGCCGCAGCACTCTA-3' |
| *Spire1* | 5'-CACATTAATGGATTTGCCAACTG-3' | 5'-ACTATTCCCTGCAGGTTGTTTACTG-3' |
| *Tmem100* | 5'-ACCCTACCGCCCTACGCTAA-3' | 5'-CCCCTCCTGGGCTGGTT-3' |
| *Tmem154* | 5'-CGCAGAGGAAGAGACGACAAG-3' | 5'-CGCGCTTTCAGCTAATGCT-3' |
| *Tnnt2* | 5'-AGTTCGACCTGCAGGAAAAGTT-3' | 5'-CCTGTTTCGCAGAACGTTGA-3' |
| *Tnrc6b* | 5'-TGCCAAATCTCCACCGACTAA-3' | 5'-GAGGCCAACTGGCATTGC-3' |
| *Trex1* | 5'-TGCTGGCTGTCCACAGACAT-3' | 5'-TGGCTGCCCCTCAGACAT-3' |
| *Ube2o* | 5'-TCTCCATCCAAGGTCTGATCCT-3' | 5'-GGCCCCGGTCACTATCAAA-3' |
| *Vcan* | 5'-ACCCTTTGACTGTCAGCAAGTTT-3' | 5'-GACTCCCGTGGCTGACATG-3' |
| *Vsx2* | 5'-TTGCTGTTGTAGCCAATTGGTT-3' | 5'-TTCGAGCTGCACTGGTTTTCT-3' |
| *Whamm* | 5'-ACAAGACCGCAGCTCATCCT-3' | 5'-AGTGGCTTCAGGTAAGGGAGAA-3' |
| *Zic1* | 5'-TTTCCCTGCCCGTTTCCT-3' | 5'-GCGTCCTTTTGTGGATCTTGA-3' |
| *Zic2* | 5'-CCACCAACGCTTGTGAATGTA-3' | 5'-CGCAAAAGCACTGAAACATCA-3' |

**Supplementary Dataset S1. List of 84 genes that were increased more than 1.5-fold from 4- to 8-week-old or 10-week-old ICR samples.** Genes whose expression level was increased more than 1.5-fold from 4- to 8-week-old or 10-week-old are indicated by "●". Numbers of differentially expressed genes indicated above “Gene name”, “8weeks”, “10weeks” column. The nine columns on the right show the signal value in each sample.

**Supplementary Dataset S2. List of 98 genes that were decreased more than 2.25-fold from 4- to 8-week-old or 10-week-old ICR samples.** Genes whose expression level was decreased more than 2.25-fold from 4- to 8-week-old or 10-week-old are indicated by "●". Numbers of differentially expressed genes indicated above “Gene name”, “8weeks”, “10weeks” column. The nine columns on the right show the signal value in each sample.

**Supplementary Dataset S3. List of 113 genes that were increased more than 1.5-fold from 4- to 8-week-old or 10-week-old SD rat samples.** Genes whose expression level was increased more than 1.5-fold from 4- to 8-week-old or 10-week-old are indicated by "●". Numbers of differentially expressed genes indicated above “Gene name”, “8weeks”, “10weeks” column. The three columns on the right show the signal value in each sample.

**Supplementary Dataset S4. List of 57 genes that were decreased more than 2.25-fold from 4- to 8-week-old or 10-week-old SD rat samples.** Genes whose expression level was decreased more than 2.25-fold from 4- to 8-week-old or 10-week-old are indicated by "●". Numbers of differentially expressed genes indicated above “Gene name”, “8weeks”, “10weeks” column. The three columns on the right show the signal value in each sample.

**Supplementary Dataset S5. List of 68 genes that were increased more than 1.5-fold from 4- to 8-week-old or 10-week-old only ICR samples.** Genes whose expression level was increased more than 1.5-fold in ICR only, compared to genes extracted in ICR and SD rat. Genes whose expression level was increased more than 1.5-fold from 4- to 8-week-old or 10-week-old are indicated by "●". Numbers of differentially expressed genes indicated above “Gene name”, “8weeks”, “10weeks” column. The nine columns on the right show the signal value in each ICR sample.

**Supplementary Dataset S6. List of 66 genes that were decreased more than 2.25-fold from 4- to 8-week-old or 10-week-old only ICR samples.** Genes whose expression level was increased more than 2.25-fold in ICR only, compared to genes extracted in ICR and SD rat. Genes whose expression level was decreased more than 2.25-fold from 4- to 8-week-old or 10-week-old are indicated by "●". Numbers of differentially expressed genes indicated above “Gene name”, “8weeks”, “10weeks” column. The nine columns on the right show the signal value in each ICR sample.

**Supplementary Dataset S7. List of 1065 mutations over moderate in ICR.** List of ICR mutation points that are MODERATE or HIGH compared to the reference sequence. Chrom indicates chromosome number, Pos indicates position, Ref_allele indicates the base in the reference sequence, Alt_allele indicates the base in the ICR, and Unique_Effect indicates what kind of mutation it is. Unique_Effect indicates what kind of mutation it is. Annotation_Impact states whether the impact of the mutation is MODERATE or HIGH. The Gene column lists the gene names.

**Supplementary Dataset S8. List of 60 mutations High in ICR.** List of ICR mutation points that are HIGH compared to the reference sequence. Chrom indicates chromosome number, Pos indicates position, Ref_allele indicates the base in the reference sequence, Alt_allele indicates the base in the ICR, and Unique_Effect indicates what kind of mutation it is. Unique_Effect indicates what kind of mutation it is. Annotation_Impact states whether the impact of the mutation is MODERATE or HIGH. The Gene column lists the gene names.
